# GWAS‐informed variant‐to‐gene mapping integration with microglial single‐cell non‐coding CRISPRi screen illuminates genetic etiology of Alzheimer’s disease

**DOI:** 10.1002/alz.095581

**Published:** 2025-01-09

**Authors:** Shannon Laub, Natalia Tulina, Sandhya Ramachandran, Svathi Murali, Louisa Boateng, J. Bormeh Faryean, Matthew Hoffman, Kieona Cook, James A. Pippin, Mitchell Conery, Elizabeth A Burton, Andrew D. Wells, Li‐San Wang, Gerard D. Schellenberg, Stewart A. Anderson, Struan F.A. Grant, Alessandra Chesi

**Affiliations:** ^1^ University of Pennsylvania, Philadelphia, PA USA; ^2^ The Children’s Hospital of Philadelphia, Philadelphia, PA USA

## Abstract

**Background:**

Recent genome‐wide association studies (GWAS) of Alzheimer’s disease (AD) have identified approximately 70 genetic loci linked to the disorder. The pivotal challenge in the post‐GWAS era is dissecting the underlying causal variants and effector genes, a crucial step for effective therapeutic development. Most of these variants reside in non‐coding regions of the genome, suggesting their regulatory role in distal gene expression.

**Method:**

Consequently, we developed a genomic approach based on high‐resolution promoter Capture‐C, ATAC‐seq and RNA‐seq plus single‐cell non‐coding CRISPR interference screening to identify candidate causal variants and their effector genes. We leveraged our collection of genomic datasets from 14 neuronal, astrocyte, and microglial cell types to prioritize candidate regulatory regions containing AD‐associated SNPs by requiring them to reside in open chromatin and contact an open gene promoter.

**Result:**

We identified 77 candidate regions that have spatial contacts with 85 putative effector genes. To validate these findings in a high‐throughput manner on a key cell type for AD, i.e. microglia, we performed a pooled enhancer CRISPRi screen at low MOI followed by scRNA‐seq in the human cell line HMC3. We designed 269 sgRNA guides targeting 77 putative enhancer regions (3 guides per region), 6 positive controls, and 22 non‐targeting controls. These were transduced as a pool using lentiviral vectors. After selection, cells were run on the 10x Chromium platform. We performed quality control and analysis of ∼ 97,000 cells using Seurat. Differential gene expression analyses between cells containing a single perturbation and cells containing a non‐targeting guide using SCEPTRE and MAST yielded 10 hits. We validated several of these leads via bulk CRISPRi and/or luciferase assays in HMC3, including an enhancer region containing three AD‐associated SNPs located in an intron of *TSPAN14*. Precise deletion of this enhancer via CRISPR/Cas9 in several clonal lines resulted in downregulation of *TSPAN14* expression, highlighting its role as a putative effector gene in AD.

**Conclusion:**

Further functional investigations are required to clarify the role of these variants and genes in AD pathogenesis. We plan to expand our screens to other cell types, and we anticipate this methodology will be portable to other neurodegenerative or neurodevelopmental disease contexts.

